# Are People Experiencing the ‘Pains of Imprisonment’ During the COVID-19 Lockdown?

**DOI:** 10.3389/fpsyg.2020.578430

**Published:** 2020-11-19

**Authors:** Mandeep K. Dhami, Leonardo Weiss-Cohen, Peter Ayton

**Affiliations:** ^1^Department of Psychology, Middlesex University, London, United Kingdom; ^2^Leeds University Business School, University of Leeds, Leeds, United Kingdom

**Keywords:** coronavirus, COVID-19, lockdown, imprisonment, psychological adjustment

## Abstract

**Background:**

By the end of March 2020, more than a fifth of the world’s population was in various degrees of “lockdown” in order to slow the spread of COVID-19. This enforced confinement led some to liken lockdown to imprisonment. We directly compared individual’s experiences of lockdown with prisoners’ experiences of imprisonment in order to determine whether psychological parallels can be drawn between these two forms of confinement.

**Methods:**

Online surveys of adults in lockdown in the UK (*N* = 300) and California (*N* = 450) were conducted 4 and 5 weeks into lockdown in each region, respectively. The UK data was then compared to Souza and Dhami’s (2010) sample of 267 medium security prisoners in England, and the Californian data was compared to Dhami et al.’s (2007) sample of 307 medium security Federal prisoners in California. We measured the effects of Group (Lockdown v. Prison) on five categories of dependent variables (i.e., activity, social contact, thoughts, feelings, and rule-breaking), controlling for demographic differences between the groups.

**Results:**

In both regions, people in lockdown thought significantly less often about missing their freedom, as well as missing their family and friends living elsewhere than did first-time prisoners. However, people in lockdown in both regions were also significantly less engaged in a range of daily activities than were first-time prisoners. Additionally, in both regions, people in lockdown reported feeling more hopeless than first-time prisoners.

**Conclusion:**

Although Governments introducing lockdown policies do not intend to punish their citizens as courts do when sending convicted offenders to prison, such policies can have unintended adverse consequences. Psychological parallels can be drawn between the two forms of confinement.

## Introduction

In January 2020, China began to “lockdown” its citizens in an effort to contain a new coronavirus (SARS-CoV-2) and slow the spread of Covid-19, the potentially fatal disease it causes. By the end of March 2020, more than a fifth of the world’s population was estimated to be in various degrees of lockdown (Gilbert, 2020). Workplaces were closed and employees either had to work from home or were out of employment. Schools and childcare facilities were also closed, as were other indoor and outdoor spaces where people may congregate and interact (e.g., restaurants, cinemas, shops and businesses selling “non-essential” goods or services, places of worship, gyms, swimming pools and playgrounds). People were only allowed to leave their homes for a limited number of essential purposes (e.g., if they were designated “key” workers, to buy food, seek medical attention, and for limited exercise). In some regions, lockdowns were police enforced and violations of the rules of lockdown could result in criminal sanctions ranging from fines to custody (e.g., Executive Department State of California, 2020; UK Government, 2020c)^1^.

The enforced confinement led some to compare the COVID-19 lockdown to imprisonment. Although the US TV host Ellen DeGeneres joked in her comparison of the two forms of confinement (Michallon, 2020), others, including ex-prisoners, have been more serious in their consideration of the similarities between the COVID-19 lockdown and imprisonment (e.g., O’Donnell, 2020; Toon, 2020; Wheatcroft, 2020). Indeed, the term “lockdown” is commonly used in the prison system and forms part of the daily regime when the movement and free association of some prisoners is controlled, and it is part of emergency procedures dealing with prisoner unrest. Clearly, Governments introducing lockdown policies to control the pandemic do not intend to punish their citizens as courts do when imposing prison sentences on convicted offenders. Nevertheless, lockdown policies may have unintended adverse consequences that are akin to the “pains of imprisonment” (e.g., Sykes, 1958; Sykes and Messinger, 1960; Goffman, 1961). Imprisonment deprives prisoners of their individual freedom. It restricts their movement and physical contact with family and friends outside. Imprisonment reduces prisoners’ access to potential heterosexual relations and some previously enjoyed goods and services. Imprisonment can also adversely affect prisoners’ sense of personal safety.

In the present paper, we directly compare individual’s experiences of the COVID-19 lockdown with prisoners’ experiences of imprisonment in order to determine if psychological parallels can be drawn between these two forms of confinement. The present research thus also sheds light on some psychological, emotional, social and behavioral responses to lockdown. Patterns of adjustment can have implications for how well people can cope with lockdown and how well they can readjust to life after lockdown. Before describing the present research, we compare features of the COVID-19 lockdown and imprisonment, and review findings of recent research on the subjective experiences of both.

### The COVID-19 Lockdown vs. Imprisonment^2^

The COVID-19 lockdown and imprisonment can be compared on several dimensions that may affect an individual’s subjective experiences. First, in terms of purpose, the COVID-19 lockdown is an extreme public health policy and imprisonment is a harsh criminal justice policy. In addition, self-isolation is obligatory for those displaying symptoms of COVID-19, whereas solitary confinement is typically reserved for those who frequently break prison rules. Both forms of confinement represent control strategies (i.e., for the virus v. crime, respectively), and both entail a component of protecting others (i.e., from being infected v. victimized) as well as reducing the burden on Government agencies and services (i.e., healthcare system v. criminal justice system).

Second, in terms of expectation and duration, unlike the COVID-19 lockdown, which may have come as a surprise to many, most offenders will know that they face imprisonment when they attend their sentencing hearing. However, both the lockdown and prison sentences typically have time limits associated with them (e.g., the 21 days lockdown in the UK which was announced on March 23rd 2020; see UK Government, 2020a), although the end to both may be uncertain and could be extended (e.g., as was widely anticipated the UK lockdown was extended for 3 weeks on April 16th 2020 and partial easing only began on May 12th 2020)^3^. Nevertheless, prison sentences are typically considerably longer, especially for serious offenders and those with previous convictions. Although not necessarily equivalent, it is becoming increasingly clear that the “shielding” of those particularly vulnerable to the more severe consequences of COVID-19 (e.g., the elderly and people with certain underlying health conditions) is likely to continue after lockdown ends, perhaps until a vaccine is available.

Third, with regard to rules and regime, there are rules for observing the COVID-19 lockdown which limit personal freedom (e.g., not going out to work unless a key worker, going out for essential activities only, social distancing, wearing facemasks, limits on outdoor exercise, etc.). However, these do not permeate through every aspect of an individual’s life as do the rules and regime in prison, especially more secure ones. Citizens retain freedom and control in terms of sleep/rest, socializing (albeit not-in-person), diet, and accessing many goods and services online. In addition, whereas the violation of prison rules can result in extra days being added onto a sentence, lockdown rules may not be enforced, and if they are, punitive measures such as imprisonment will be rare.

Finally, in terms of living conditions, people in the COVID-19 lockdown may live alone or share their home with family or friends. Although these may sometimes be overcrowded, noisy surroundings, lacking in privacy, as is typical in prison environments, the conditions are unlike prison. This is because prisoners are “housed” in cells (e.g., typically one room with a bed, toilet and washbasin), and they may share this with someone unknown to them. Prisoners may have a fear of attack by other inmates. Similarly, it is becoming clear that domestic abuse has risen during lockdown and that some victims may be unable to seek help (e.g., ABC News, 2020; BBC News, 2020).

Thus, while it may seem apt to compare the COVID-19 lockdown to imprisonment in some respects, it does not in many others. Nevertheless, the subjective experiences of both forms of confinement may not differ so much. There is emerging evidence of individuals’ psychological, emotional, social and behavioral adjustment to lockdown, and this can be considered in light of research on prisoners’ adjustment to imprisonment.

### Review of Research on Experiences of Lockdown and Imprisonment

Brooks et al. (2020) reviewed 24 studies published between 2003 and 2018 examining the psychological effects of quarantine. Only a handful of these compared individuals who had been quarantined to those who had not. Studies with no comparison group generally reported a high prevalence of symptoms of psychological distress and disorder. In support of these findings, studies with comparison groups found higher levels of stress disorders and post-traumatic stress symptoms in those who had been quarantined, both immediately afterward and sometimes at least as much as 3 years later. Depression was also apparent for several years afterward in those who had been quarantined compared to those who had not. There was some evidence that increased duration of time in quarantine was associated with poorer mental wellbeing. Finally, the physical confinement, absence of usual routine, lack of social contact, and inability to access goods and services, resulted in frustration, boredom and feelings of isolation.

Emerging research on adults’ experiences of the COVID-19 lockdown in regions including Italy, Spain, Bangladesh, India, and the UK, has generally found high levels of stress, depression and anxiety and/or low levels of mental wellbeing (Ali et al., 2020; Aymerich-Franch, 2020; Odriozola-Gonzalez et al., 2020; Rossi et al., 2020; Srilakshmidevi and Suseela, 2020; White and Van der Boor, 2020). In addition, Aymerich-Franch (2020) found that over half of adults in lockdown in Spain felt more trapped than before lockdown, and 70.2% felt less free. Adults in lockdown in India reported disruption in their daily routine and social contacts (Sharma and Subramanyam, 2020), and whereas those in lockdown in Spain and Zimbabwe reported an increased frequency of doing household chores (Aymerich-Franch, 2020; Chirombe et al., 2020) and use of media/social media (Aymerich-Franch, 2020), those in lockdown in India reported spending most of their time resting, and not engaging in physical exercise (Singhal and Vijayaraghavan, 2020).

However, much of the aforementioned research lacks comparison groups to benchmark these reported levels of experiences. In one notable exception, Sibley et al. (2020) compared the mental and physical health and subjective wellbeing of adults during the first 18 days of lockdown in New Zealand and a matched sample of adults a few months before lockdown, as well as the same participants a year earlier. Both the between- and within-subjects analyses showed an effect of lockdown on some measures (e.g., after lockdown there was increased mental distress, sense of community and decreased fatigue). There was, however, no effect of lockdown on other measures (e.g., rumination, subjective health, perceived social support, satisfaction with health, and personal relationships). This suggests that while lockdown may lead to increased mental distress in people compared to others and themselves before lockdown, individuals may be resilient in other ways.

Finally, in another effort to contextualize and interpret the effects of lockdown, Ali et al. (2020) reported a *post hoc* comparison of the mental wellbeing of adults in lockdown in Bangladesh measured using the Warwick Edinburgh Mental Wellbeing Scale with the wellbeing scores reported by others using the same scale. They found that scores for their lockdown sample were lower than for the general population in Brazil, Denmark, England and Spain; lower than bereaved carers in the UK; lower than university students in China; and lower than healthcare workers in Northern Ireland and in Pakistan. However, the scores were similar to that of primary healthcare patients in hospital in Norway. In addition, and of particular relevance to the present study, Ali et al.’s (2020) lockdown sample scored similar to Tweed et al.’s (2018) sample of Scottish prisoners. The lockdown sample had a mean score of 38.4 (*SD* = 11.3) whereas prisoners had a mean score ranging from 37.4 (*SD* = 12.0) for remand prisoners and 41.2 (*SD* = 12.3) for other prisoners. Thus, Ali et al.’s (2020) study suggests that the COVID-19 lockdown may be associated with very similar levels of poor mental wellbeing in ordinary citizens as that seen in prison populations.

It is possible to compare the experiences of those in lockdown and those in prison because the questions that prison researchers have asked about inmates’ experiences of confinement are akin to those asked in recent COVID-19 lockdown studies. For instance, as illustrated below, researchers have measured prisoners’ participation in prison regime activities, their compliance with prison rules, their social contact with others in prison as well as with family and friends from outside, their thoughts about missing freedom and needing control over their lives, and missing heterosexual relations, and their psychological wellbeing. Prison studies can shed some light on how people might adjust to life in lockdown as well as afterward.

Evidence suggests that prisoners may suffer from adverse psychological wellbeing on measures such as depression and stress (e.g., Edwards and Potter, 2004; Van Harreveld et al., 2007; Tweed et al., 2018), and they are more likely to commit suicide than non-incarcerated populations (Fazel et al., 2017). Education, work, exercise, faith-based activities, and rehabilitative or self-help programs can provide opportunities for a constructive and stimulating use of time inside prison. Similarly, prison visitations by family and friends help maintain contact with the outside world, and social interactions with other inmates facilitate survival inside. Research suggests that participation in regime activities and having social ties can improve psychological well-being in prison, reduce misconduct in prison, and increase the chances of post-release success (e.g., Cecil et al., 2000; Camp et al., 2008; Cashin et al., 2008; Richmond, 2014; Brunton-Smith and McCarthy, 2016; Brazão et al., 2018; Kyprianides and Easterbrook, 2020). However, not all prisoners can, or do, participate in the full range of activities offered in prison, and it can be difficult to maintain meaningful relationships with family and friends outside prison as well as have supportive interactions with those inside (e.g., Dhami et al., 2007; Souza and Dhami, 2010). In a recent study of short-term imprisonment in the UK, O’Connor et al. (2020, p. 3) reported that, over time, prisoners moved “toward feeling ever more trapped or ‘banged up’.”

## The Present Research

In order to directly compare individuals’ experiences of the COVID-19 lockdown with prisoners’ experiences of imprisonment we conducted two online surveys of adults in lockdown—one in the UK, and the other in California, United States. We then compared the responses of these two samples to those reported by prisoners in medium security prisons in their respective regions. We chose the UK and California regions primarily because we had access to relevant prison data from these regions. The prison data was collected years before the current pandemic (i.e., Dhami et al., 2007; Souza and Dhami, 2010) and so is not confounded by the current COVID-19 outbreak in the prison system (California Department of Corrections and Rehabilitation, 2020; UK Government, 2020b).

There are tangible and intangible similarities and differences between the UK and California (see Business Insider, 2018; Office for National Statistics, 2020; United States Census Bureau, 2020). For instance, the UK has a population of approximately 67 million compared to approximately 40 million in California. Before the pandemic, California’s economy was slightly larger than the UK. Those aged over 65 and those from non-White racial/ethnic groups are considered to be particularly vulnerable to the harmful effects of Covid-19. The relative proportions of over 65-year-olds is 18% and 15% in the UK and California, respectively, and 14% of the UK population is non-White compared to 28% in California. The COVID-19 lockdown required people to stay at home, which also necessitated greater use of the internet. The average household size is 2.4 in the UK compared to 2.9 in California, and 96% of households in the UK had internet connection, while 85% in California had broadband internet subscription. It is worth noting that the UK has a publically funded healthcare system, whereas 9% (of those aged under 65) in California have no health insurance. The first confirmed case of COVID-19 in California was January 26th 2020 and February 21st 2020 in the UK, with the first deaths in February 6th and March 6th of the same year, respectively. The lockdown in both regions began around the same time (i.e., March 20th and 24th 2020, respectively). The present study is focused on the comparison between the lockdown and prison samples in each region, but those interested in comparing people in lockdown across the two regions can look at the regional comparison analysis which is part of the online Supplementary Materials^4^. Suffice it to say, there were very few differences between the two lockdown samples.

Specifically, we aimed to compare the responses of the two groups (i.e., lockdown and prison) on a range of behaviors, thoughts and feelings as follows: (1) total number/variety of *activities* participated in; (2) *social contact* with others in prison/living space and with those from the outside; (3) *thoughts* about missing freedom, needing control over life, missing sex, missing family and/or friends, and being attacked/beaten up; (4) *feelings* of happiness and hopelessness relative to before prison/lockdown; and (5) *rule-breaking* in prison/during lockdown. The lack of prior research on this topic led us to use non-directional hypothesis tests, with corrections for multiple tests. The present research was registered with the Open Science Framework (OSF)^5^, and the raw data and research script are available online (see footnote 4).

## Materials and Methods

### Participants

We conducted an online survey of 300 adults in lockdown in the UK and 450 adults in lockdown in California. Data was collected in the UK from April 20th to 22nd and in California from April 24th to 28th. Thus, data collection occurred approximately 4 and 5 weeks after lockdown began in each region, respectively. As mentioned, the UK data was compared to Souza and Dhami’s (2010) sample of 267 prisoners from two medium security prisons in England. The Californian data was compared Dhami et al.’s (2007) 307 prisoners from a medium security Federal prison in California, who had provided information on their prior prison experience, which as will be seen below, is pertinent for present purposes^6^. The size of the two lockdown samples were larger than their respective prison samples due to oversampling in case of low response rates or missing data, and for the potential need to conduct analyses by subgroup (see “Analysis” section below). Table 1 presents the demographic characteristics of the two lockdown samples and two prison samples.

**TABLE 1:** Demographic characteristics of UK and Californian lockdown and prison samples (% unless otherwise stated).

	UK		California	
	Lockdown (*N* = 300)	Prison (*N* = 267)	Lockdown (*N* = 450)	Prison (*N* = 307)
Female^*a*^	63.5	0.0	48.0	0.0
Age^+^	33.07 (11.14)	32.42 (11.89)	30.62 (11.22)	37.36 (10.37)
Ethnicity—BAME^*b*^	13.0	23.2	56.9	69.3
Not finish high school	0.7	47.4	1.1	32.9
Unemployed	12.8	30.5	15.8	8.2
Not in relationship	65.0	24.4	60.6	15.5
Used drugs before^*c*^	8.0	64.6	8.3	58.2
In prison before	0.3	61.8	1.1	35.8
Days inside^+*d*^	30.17 (7.79)	788.29 (1316.22)	37.66 (9.63)	1306.36 (1442.67)
Quality of life before^+*e*^	2.26 (1.29)	0.65 (2.12)	2.28 (1.34)	1.65 (1.78)

*^+^Mean (SD).*

*^*a*^In addition to the remainder who were males, some participants identified as “other” (UK lockdown: n = 1 and US lockdown: n = 7).*

*^*b*”^Black, Asian and Minority Ethnic” refers to all non-white categories.*

*^*c*^This variable was made binary and the data in this table refer to responses of “sometimes” (4) to “often” (7) on the rating scale.*

*^*d*^“Days inside” refers to the number of days in lockdown or prison for the current sentence.*

*^*e*^Following Dhami et al. (2007), we computed a “quality-of-life-before” lockdown/prison variable. Here, this represents an aggregate of education (did not finish high school v. did), work (unemployed v. employed/other), relationship status (alone v. relationship), and used drugs before (yes v. no). A positive response was coded as 1 and a negative as -1 (reverse coding was employed for the drugs variable).*

### Survey

The survey called “Life in Lockdown” comprised six sections (i.e., Your Life in Lockdown, Socializing with Others, Your Health, Rules of Lockdown, Your Experiences of Covid-19, and You and Your Life Before Lockdown). It included items adapted from Dhami et al. (2007) and although there were some necessary differences to item wording, item order and response scales remained the same (see Supplementary Table A1). In addition, we included an item asking participants how similar they thought lockdown was to imprisonment, to which responses were provided on a “very different” 1 to “very similar” 7-point scale labeled at each end. Analyses of the remaining survey items without any comparison to prison data are in preparation and will be reported by the authors elsewhere (the full survey can be found online, see footnotes 4 and 5).

For present purposes, and akin to Dhami et al.’s (2007) study on adaptation to imprisonment, the items include five categories of 11 dependent variables^7^ :

(1) Activity (i.e., total number/variety of activities participated—work^8^, education, exercise, religion and self-help programs);
(2) Social contact (i.e., amount of interaction with others in prison/living space, and frequency of contact with family and/or friends from the outside^9^);
(3) Thoughts (i.e., frequency of thoughts about missing freedom, needing control over life, missing sex, missing family and/or friends, and being attacked/beaten up);
(4) Feelings (i.e., degree of happiness and hopelessness relative to before prison/lockdown); and
(5) Rule-breaking^10^ (i.e., frequency of charges of misconduct in prison/accusations of disobeying rules of lockdown).

### Procedure

Participants in the lockdown samples were recruited online using Prolific Academic. Only individuals who reported being fluent in English and currently residing in the UK or the US state of California were allowed to participate. Each participant was paid £2 (or its USD equivalent) for completing the survey. Participants could skip questions, but could not go back to change previous answers. Ethical approval for the research was granted by the Research Ethics Committee at the Department of Psychology, City, University of London.

According to Dhami et al. (2007) and Souza and Dhami (2010), prisoners were randomly selected from the prison roll. Their participation was voluntary and anonymous but not compensated. The survey was self-administered in groups in the education or chapel areas of the prisons and in the absence of prison staff. Interpreters were provided for Hispanic-only speaking prisoners in California, and the trained researchers administered the survey individually to prisoners who reported having difficulties in reading and/or writing.

### Data Analysis

Data analysis occurred in three steps, with the first two being preliminary analyses. First, it is clear from Table 1 that, whereas participants in the two lockdown samples included both males and females, all prisoners were in male prisons. In addition, while the vast majority of participants in the lockdown samples had never been to prison before, a sizeable proportion of participants in the prison samples had prior prison experience (i.e., were recurrent). Thus, preliminary analyses using multivariate analysis of variance tests (MANOVA) were conducted to determine whether the male and female groups in each of the two lockdown samples and the first-time and recurrent prisoners in each of the two prison samples should be grouped together or not for the main analysis. Here, gender (i.e., male v. female) and prison experience (i.e., first-time v. recurrent) were the independent variables in the respective analyses, and the dependent variables were the 11 listed earlier. These tests were performed for each sample and region separately (i.e., UK lockdown, UK prison, Californian lockdown, Californian prison).

Second, we compared the groups to be examined in the main analysis on the remaining demographic characteristics (see Table 1) using a combination of independent samples *t*-tests and Chi-Square tests. The comparisons in this preliminary analysis were conducted for each region separately, and helped to identify variables to be controlled in the main analysis.

Finally, in order to fulfill the main aim of the present research i.e., to determine if experiences of the COVID-19 lockdown were similar or different from imprisonment, we conducted Analysis of covariance (ANCOVA) and multivariate analysis of covariance (MANCOVA) tests. These measured the effects of group (Lockdown v. Prison) on the five categories (i.e., activity, social contact, thoughts, feelings, and rule-breaking) of 11 dependent variables, controlling for any differences in demographic characteristics as covariates in the analysis. As mentioned, the present study is focused on the comparison between the lockdown and prison samples in each region (i.e., the UK and California), and so the tests were performed for each region separately. Holm’s (1979) method was used to correct for multiple comparisons when reporting univariate test results from the MANCOVA analyses.

## Findings

The mean rating of how similar lockdown was to imprisonment was 2.28 (*SD* = 1.49) for participants in lockdown in the UK and 2.11 (*SD* = 1.46) for participants in lockdown in California. There was no significant difference between the two regions, *t*(748) = 1.55, *p* = 0.121.

### Preliminary Analyses

The preliminary MANOVAs across all the 11 dependent variables revealed a significant multivariate effect of gender (male v. female) for both the UK and the Californian lockdown samples [UK: *F*(11, 253) = 4.14, *p* < 0. 001, η*_*p*_*^2^ = 0.15 and Californian: *F*(11, 351) = 4.09, *p* < 0. 001, η*_*p*_*^2^ = 0.11]^11^. There were overall gender differences in the responses of males and females. Therefore, we split the lockdown samples by gender in further analyses.

There was also a significant multivariate effect of prison experience (first-time v. recurrent) for both the UK and Californian prison samples [UK: *F*(11, 224) = 7.66, *p* < 0.001, η*_*p*_*^2^ = 0.27 and Californian: *F*(11, 255) = 2.02, *p* = 0.027, η*_*p*_*^2^ = 0.08]. First-time prisoners gave different responses overall than did recurrent prisoners. Given that the lockdown samples were experiencing their first ever lockdown experience, we thought it would be reasonable to compare their responses with the first-time prisoners in each region, thereby excluding the recurrent prisoners from further analyses.

Next, as Table 2 shows, the independent samples *t*-tests and Chi-Square tests revealed several differences in each region between the demographic characteristics of males (and females) in lockdown and first-time prisoners. Thus, we will control for these variables in the main analysis.

**TABLE 2:** Demographic characteristics of males and females in lockdown and first-time prisoners (% unless otherwise stated).

	UK			California		
	Lockdown Males (*N* = 107)	First-time Prisoners (*N* = 102)	Lockdown Females (*N* = 190)	Lockdown Males (*N* = 220)	First-time Prisoners (*N* = 197)	Lockdown Females (*N* = 216)
Age^+^	32.35** (11.09)	37.79 (14.74)	33.41** (11.21)	29.96*** (10.77)	36.23 (10.24)	31.09*** (11.43)
Ethnicity—BAME	8.4***	30.7	15.3**	61.4*	71.2	52.3***
Not finish high school	0.0***	25.0	1.1***	1.4***	32.1	0.9***
Unemployed	12.3	21.8	13.2	13.2	8.0	18.1**
Not in relationship	70.1***	23.2	62.6***	67.3***	12.1	54.0***
Used drugs before	15.9**	34.0	3.7***	7.3***	53.2	7.4***
Days inside^+^	29.44*** (10.07)	798.32 (1186.21)	30.62*** (6.16)	37.11*** (9.92)	1237.08 (1267.10)	38.17*** (9.34)
Quality of life before^+^	2.03 (1.43)	1.84 (1.66)	2.38** (1.19)	2.21* (1.38)	1.84 (1.77)	2.39*** (1.31)

^+^Mean (*SD*). Comparisons are for males in lockdown v. first-time prisoners, females in lockdown v. first-time prisoners. ***p < 0.001, **p < 0.01, *p < 0.05.

### Comparison of Life in COVID-19 Lockdown and Prison in the UK

In order to determine if experiences of the COVID-19 lockdown were similar or different from first-time prisoners’ experiences of imprisonment, we conducted ANCOVAs and MANCOVAs on the five categories (i.e., activity, social contact, thoughts, feelings, and rule-breaking) of 11 dependent variables. Group (Lockdown or Prison) was the independent variable and age, ethnicity (White or BAME), quality of life before, and time inside (Short or Long)^12^ were entered as covariates in the analyses. Separate analyses were conducted for males and females in lockdown. Here, we describe the results for the Group variable. Later, we return to a consideration of the effects of any statistically significant covariates. Table 3A presents the means and standard deviations of the 11 dependent variables by Group for the UK sample.

**TABLE 3A:** Means and standard deviations of dependent variables by group (UK Sample).

	Males in lockdown		First-time prisoners		Females in lockdown	
	*M*	*SD*	*M*	*SD*	*M*	*SD*
Activity	1.98	1.07	2.83	1.24	2.02	1.07
Social contact						
Those inside	5.52	1.36	5.15	1.58	5.85	1.45
Those outside	6.07	1.78	6.25	1.99	6.56	1.53
Thoughts						
Freedom	5.50	1.98	7.65	1.72	5.89	2.06
Control	5.60	2.24	6.21	2.41	6.04	2.24
Sex	4.45	2.78	6.27	2.38	3.37	2.55
Family/friends	5.91	2.02	8.23	1.49	7.01	1.73
Attack	1.88	1.44	2.38	1.94	1.99	1.65
Feelings						
Happiness	–0.64	1.49	–1.36	2.16	–0.67	1.71
Hopelessness	0.36	1.56	–0.66	2.69	0.39	1.97
Rule-breaking	1.55	1.15	1.57	1.09	1.23	0.72

#### Activity

An ANCOVA comparing males in lockdown and first-time prisoners found that after controlling for a significant effect of age (*p* < 0.001, for all other covariates *p*s > 0.05)^13^, Group had a significant effect on the total number or variety of activities that participants took part in i.e., work, education, exercise, religion and self-help programs, *F*(1, 200) = 32.47, *p* < 0.001, η*_*p*_*^2^ = 0.14. As Table 3A shows, males in lockdown participated in fewer activities, on average, than did first-time prisoners.

The ANCOVA comparing females in lockdown and first-time prisoners similarly showed a significant effect of Group on activity [*F*(1, 283) = 38.61, *p* < 0.001, η*_*p*_*^2^ = 0.12], after controlling for a significant effect of age (*p* = 0.005) and quality of life before (*p* = 0.004). Like their male counterparts, females in lockdown participated in, on average, fewer activities than first-time prisoners (see Table 3A).

#### Social Contact

When comparing males in lockdown and first-time prisoners, the correlation among the two measures of social contact (i.e., amount of interaction with others in prison/living space, and frequency of contact with family/friends from the outside) was *r* = 0.08. A MANCOVA revealed that after controlling for significant effects of age (*p* = 0.002) and ethnicity (*p* = 0.034), there was a significant multivariate effect of Group on social contact, *F*(2, 189) = 4.38, *p* = 0.014, η*_*p*_*^2^ = 0.04. However, the univariate *F*-tests found no significant effect of Group on either the amount of interaction with others in prison/living space [*F*(1, 190) = 3.63, *p* = 0.084, η*_*p*_*^2^ = 0.02] or the frequency of contact with family/friends from the outside, *F*(1, 190) = 4.20, *p* = 0.084, η*_*p*_*^2^ = 0.02. Males in lockdown, thus, have similar levels of interaction with those in their living space and a similar frequency of contact with family/friends from the outside as first-time prisoners.

For the analyses comparing females in lockdown and first-time prisoners, the correlation between the two measures of social contact was *r* = 0.19. After controlling for significant effects of age (*p* = 0.006), ethnicity (*p* < 0.001), and time inside (*p* = 0.035), there was a significant effect of Group on social contact, *F*(2, 262) = 7.23, *p* < 0.001, η*_*p*_*^2^ = 0.05. The univariate *F*-tests showed a significant effect of Group on the amount of interaction with others in prison/living space [*F*(1, 263) = 13.39, *p* < 0.001, η*_*p*_*^2^ = 0.05], but not on frequency of contact with family/friends from the outside, *F*(1, 263) = 0.18, *p* = 0.672, η*_*p*_*^2^ = 0.001. Thus, although females in lockdown had more interaction with those in their living space, on average, compared to first-time prisoners, females had a similar frequency of contact with family/friends from the outside as did first-time prisoners.

#### Thoughts

When comparing males in lockdown and first-time prisoners, the correlations among participants’ ratings of the frequency of their thoughts about missing their freedom, needing control over their life, missing sex, missing their family/friends, and being attacked/beaten up ranged from *r* = 0.07 to 0.62. A MANCOVA revealed that age was a significant covariate (*p* = 0.001), and after controlling for this, there was a significant effect of Group on thoughts, *F*(5, 189) = 23.48, *p* < 0.001, η*_*p*_*^2^ = 0.38. Group had a significant effect on all of the five thought variables [freedom: *F*(1, 193) = 55.07, *p* < 0.001, η*_*p*_*^2^ = 0.22; control: *F*(1, 193) = 6.52, *p* = 0.023, η*_*p*_*^2^ = 0.03; sex: *F*(1, 193) = 25.76, *p* < 0.001, η*_*p*_*^2^ = 0.12; and family/friends: *F*1, 193) = 82.37, *p* < 0.001, η*_*p*_*^2^ = 0.30; attacked: *F*(1, 193) = 5.35, *p* = 0.023, η*_*p*_*^2^ = 0.03]. On average, compared to first-time prisoners, males in lockdown thought less often about missing their freedom, needing control over their life, missing sex, missing family/friends, and being attacked/beaten up.

When comparing females in lockdown and first-time prisoners, the correlations among the thoughts variables ranged from *r* = 0.06 to 0.67. A MANCOVA showed that age was a significant covariate (*p* < 0.001), and after controlling for this, Group had a significant effect on thoughts, *F*(5, 270) = 29.31, *p* < 0.001, η*_*p*_*^2^ = 0.35. This time, Group had a significant effect on three of the thought variables [freedom: *F*(1, 274) = 40.65, *p* < 0.001, η*_*p*_*^2^ = 0.13; sex: *F*(1, 274) = 97.92, *p* < 0.001, η*_*p*_*^2^ = 0.26; and family/friends: *F*(1, 274) = 30.91, *p* < 0.001, η*_*p*_*^2^ = 0.10]. There was no significant effect of Group on the remaining two thought variables [control: *F*(1, 274) = 0.92, *p* = 0.346, η*_*p*_*^2^ = 0.003 and attacked: *F*(1, 274) = 1.87, *p* = 0.346, η*_*p*_*^2^ = 0.01]. Females in lockdown thought, on average, less often about missing their freedom, missing sex and missing family/friends than did first-time prisoners, but thought equally often about needing control over their life and being attacked/beaten up.

#### Feelings

For the comparison of males in lockdown and first-time prisoners, the correlation between feelings of happiness and hopelessness was *r* = −0.24. A MANCOVA revealed a significant multivariate effect of Group on feelings, *F*(2, 194) = 10.65, *p* < 0.001, η*_*p*_*^2^ = 0.10. None of the covariates were statistically significant, all *p*s > 0.05. According to the univariate *F*-tests, Group had a significant effect on both happiness [*F*(1, 195) = 6.90, *p* = 0.013, η*_*p*_*^2^ = 0.03] and hopelessness, *F*(1, 195) = 7.64, *p* = 0.013, η*_*p*_*^2^ = 0.04. Relative to before lockdown/prison, males in lockdown were, on average, less unhappy compared to first-time prisoners. However, males in lockdown felt more hopelessness, on average, than did first-time prisoners.

The analyses comparing females in lockdown and first-time prisoners yielded a similar pattern of results. Here, the correlation between happiness and hopelessness was *r* = −0.34. As above, there was no significant effect of any of the covariates (all *p*s > 0.05), and the effect of Group on feelings was significant, *F*(2, 277) = 17.80, *p* < 0.001, η*_*p*_*^2^ = 0.12. Again, the effect of Group was significant for both happiness [*F*(1, 278) = 8.58, *p* = 0.004, η*_*p*_*^2^ = 0.03] and hopelessness, *F*(1, 278) = 12.87, *p* < 0.001, η*_*p*_*^2^ = 0.04. Similar to their male counterparts, relative to before, females in lockdown were, on average, feeling less unhappy but more hopeless than first-time prisoners.

#### Rule-Breaking

Finally, an ANCOVA comparing males in lockdown and first-time prisoners found no significant effect of Group on the frequency of accusations of disobeying rules of lockdown/charges of misconduct in prison, *F*(1, 200) = 0.01, *p* = 0.922, η*_*p*_*^2^ = 0.000. Age (*p* = 0.002) and quality of life before (*p* = 0.043) were significant covariates in this analysis. On average, males in lockdown were equally often accused of disobeying the rules of lockdown as were first-time prisoners in being charged with misconduct in prison.

By contrast, the comparison between females in lockdown and first-time prisoners, yielded a significant effect of Group on rule-breaking [*F*(1, 283) = 9.07, *p* = 0.003, η*_*p*_*^2^ = 0.003], after controlling for a significant effect of age (*p* < 0.001) and quality of life before (*p* = 0.008). Females in lockdown were, on average, less likely to be accused of disobeying the rules of lockdown than were first-time prisoners charged with misconduct in prison.

### Comparison of Life in COVID-19 Lockdown and Prison in California

Table 3B presents the means and standard deviations of the 11 dependent variables by Group for the Californian sample.

**TABLE 3B:** Means and standard deviations of dependent variables by group (Californian sample).

	Males in lockdown		First-time prisoners		Females in lockdown	
	*M*	*SD*	*M*	*SD*	*M*	*SD*
Activity	2.17	1.13	3.58	1.13	2.12	1.16
Social contact						
Those inside	5.12	1.59	4.26	1.64	5.56	1.56
Those outside	5.40	1.72	5.35	1.98	5.81	1.78
Thoughts						
Freedom	5.23	2.30	7.98	1.73	5.70	2.27
Control	5.13	2.41	6.58	2.66	5.95	2.27
Sex	4.21	2.88	7.57	1.83	3.37	2.49
Family/friends	5.54	2.37	8.31	1.49	5.86	2.38
Attack	2.29	1.78	2.65	2.24	2.32	1.74
Feelings						
Happiness	–0.52	1.54	–0.99	2.47	–0.66	1.74
Hopelessness	0.02	1.70	–1.14	2.51	0.38	1.99
Rule-breaking	1.69	1.32	1.75	1.21	1.59	1.15

#### Activity

After controlling for significant effects of ethnicity (*p* = 0.020) and time inside (*p* = 0.037), an ANCOVA comparing males in lockdown and first-time prisoners found a significant effect of Group on the total number or variety of activities that participants engaged in, *F*(1, 400) = 143.32, *p* < 0.001, η*_*p*_*^2^ = 0.26. As Table 3B shows, males in lockdown participated in, on average, fewer activities than did first-time prisoners.

The ANCOVA comparing females in lockdown and first-time prisoners similarly showed a significant effect of Group on activity [*F*(1, 395) = 131.70, *p* < 0.001, η*_*p*_*^2^ = 0.25], after controlling for significant effects of ethnicity (*p* = 0.021) and time inside (*p* < 0.001). Like their male counterparts, females in lockdown participated in, on average, fewer activities than did first-time prisoners (see Table 3B).

#### Social Contact

When comparing males in lockdown and first-time prisoners, the correlation among the two measures of social contact was *r* = 0.14. A MANCOVA revealed a significant multivariate effect of Group on social contact, *F*(2, 353) = 8.94, *p* < 0.001, η*_*p*_*^2^ = 0.05. None of the covariates were statistically significant, all *p*s > 0.05. The univariate *F*-tests showed a significant effect of Group on the amount of interaction with others in prison/living space [*F*(1, 354) = 17.68, *p* < 0.001, η*_*p*_*^2^ = 0.05], but not on the frequency of contact with family/friends from the outside, *F*(1, 354) = 0.01, *p* = 0.938, η*_*p*_*^2^ = 0.000. Therefore, although males in lockdown had, on average, more interaction with those in their living space than did first-time prisoners, both groups had a similar frequency of contact with family/friends from the outside.

For the analyses comparing females in lockdown and first-time prisoners, the correlation between the two measures of social contact was *r* = 0.11. After controlling for a significant effect of quality of life before (*p* = 0.038), there was a significant effect of Group on social contact, *F*(2, 365) = 26.56, *p* < 0.001, η*_*p*_*^2^ = 0.13. The effect of Group was significant for both the amount of interaction with others in prison/living space [*F*(1, 366) = 50.97, *p* < 0.001, η*_*p*_*^2^ = 0.12] and on frequency of contact with family/friends from the outside, *F*(1, 366) = 3.91, *p* = 0.049, η*_*p*_*^2^ = 0.01. On average, compared to first-time prisoners, females in lockdown had more interaction with those in their living space and had a higher frequency of contact with family/friends from the outside.

#### Thoughts

When comparing males in lockdown and first-time prisoners, the correlations among the five thoughts variables ranged from *r* = 0.12 to 0.67. After controlling for significant effects of ethnicity (*p* = 0.047) and quality of life before (*p* = 0.032), a MANCOVA revealed a significant effect of Group on thoughts, *F*(5, 380) = 58.69, *p* < 0.001, η*_*p*_*^2^ = 0.44. Here, Group had a significant effect on all of the thought variables [freedom: *F*(1, 384) = 169.45, *p* < 0.001, η*_*p*_*^2^ = 0.31; control: *F*(1, 384) = 32.62, *p* < 0.001, η*_*p*_*^2^ = 0.08; sex: *F*(1, 384) = 156.28, *p* < 0.001, η*_*p*_*^2^ = 0.29; family/friends: *F*(1, 384) = 162.13, *p* < 0.001, η*_*p*_*^2^ = 0.30; and attacked: *F*(1, 384) = 5.83, *p* = 0.016, η*_*p*_*^2^ = 0.02]. On average, males in lockdown thought less often about missing their freedom, needing control over their life, missing sex, missing family/friends, and being attacked/beaten up, than did first-time prisoners.

When comparing females in lockdown and first-time prisoners, the correlations among the thoughts variables ranged from *r* = 0.03 to 0.69. A MANCOVA showed a significant effect of Group on thoughts, *F*(5, 376) = 78.68, *p* < 0.001, η*_*p*_*^2^ = 0.51. None of the covariates were statistically significant, all *p*s > .05. Group had a significant effect on all but one of the thought variables [freedom: *F*(1, 380) = 122.94, *p* < 0.001, η*_*p*_*^2^ = 0.24; control: *F*(1, 380) = 10.56, *p* = 0.003, η*_*p*_*^2^ = 0.03; sex: *F*(1, 380) = 311.51, *p* < 0.001, η*_*p*_*^2^ = 0.45; family/friends: *F*(1, 380) = 134.52, *p* < 0.001, η*_*p*_*^2^ = 0.26). The exception was thinking about being attacked/beaten up, *F*1, 380) = 3.81, *p* = 0.052, η*_*p*_*^2^ = 0.01. Like their male counterparts, females in lockdown thought, on average, less often about missing their freedom, needing control over their life, missing sex, and missing family/friends than did first-time prisoners. However, females in lockdown thought about being attacked/beaten up as equally often as did first-time prisoners.

#### Feelings

For the comparison of males in lockdown and first-time prisoners, the correlation between feelings of happiness and hopelessness was *r* = −0.21. A MANCOVA revealed that after controlling for significant effects of ethnicity (*p* = 0.039) and time inside (*p* = 0.024), there was a significant effect of Group on feelings, *F*(2, 388) = 14.87, *p* < 0.001, η*_*p*_*^2^ = 0.07. The univariate *F*-tests indicated that Group had a significant effect on both happiness [*F*(1, 389) = 5.60, *p* = 0.018, η*_*p*_*^2^ = 0.01] and hopelessness, *F*(1, 389) = 17.10, *p* < 0.001, η*_*p*_*^2^ = 0.04. As Table 3B shows, relative to before lockdown/prison, males in lockdown are, on average, feeling less unhappy, but also more hopeless, compared to first-time prisoners.

For the analyses comparing females in lockdown and first-time prisoners, the correlation between happiness and hopelessness was *r* = −0.18. After controlling for significant effects of age (*p* = 0.001) and time inside (*p* = 0.007), there was a significant effect of Group on feelings, *F*(2, 383) = 15.27, *p* < 0.001, η*_*p*_*^2^ = 0.07. However, the effect of Group was non-significant for happiness [*F*(1, 384) = 3.31, *p* = 0.069, η*_*p*_*^2^ = 0.01] and significant for hopelessness, *F*(1, 384) = 22.07, *p* < 0.001, η*_*p*_*^2^ = 0.05. Thus, relative to before, females in lockdown had similar levels of unhappiness compared to first-time prisoners, and they felt more hopeless than first-time prisoners.

#### Rule-Breaking

Finally, an ANCOVA comparing males in lockdown and first-time prisoners found no significant effect of Group on the frequency of accusations of disobeying rules of lockdown/charges of misconduct in prison, *F*(1, 401) = 1.76, *p* = 0.189, η*_*p*_*^2^ = 0.004. Age was a significant covariate in this analysis, *p* = 0.034. Therefore, on average, males in lockdown were equally often accused of disobeying the rules of lockdown as were first-time prisoners in being charged with misconduct in prison.

Similarly, for the comparison between females in lockdown and first-time prisoners, the effect of Group on rule-breaking was non-significant, *F*(1, 396) = 3.08, *p* = 0.080, η*_*p*_*^2^ = 0.01. Time inside was a significant covariate in this analysis, *p* = 0.041. As above, females in lockdown were, on average, equally likely to be accused of disobeying the rules of lockdown as were first-time prisoners charged with misconduct in prison.

### Effects of Covariates

None of the covariates demonstrated a statistically significant effect across all five categories of dependent variables either for the UK sample or the Californian sample. However, for the UK sample, age was a significant covariate in the analyses involving four of the categories (the exception was the feelings) for both comparisons between first-time prisoners and males and females in lockdown. Thus, age deserves further consideration.

Specifically, on average, older participants confined (either in lockdown or prison) in the UK were less likely than their younger counterparts to participate in activities such as work, education, exercise, religion and self-help programs. Older participants were also less likely to have contact with family/friends from outside. However, older male participants were more likely to interact with others they live with than younger male participants. Older participants were less likely than their younger counterparts to think about needing control over their life and miss sex, and they were less likely to be charged with or be accused of rule-breaking.

## Discussion

The COVID-19 lockdown resulted in the removal of individual freedoms and restrictions on movement and physical contact with family and friends who live elsewhere, as well as reduced access to potential sexual relations, some previously enjoyed goods and services, and, for some people, a sense of threat to personal safety. These are the sorts of deprivations suffered by prisoners that have been long identified in the literature on imprisonment (e.g., Sykes, 1958; Sykes and Messinger, 1960; Goffman, 1961). It is perhaps no surprise therefore, that some have likened the lockdown to imprisonment (e.g., Ali et al., 2020; O’Donnell, 2020; Toon, 2020; Wheatcroft, 2020).

In the present research, we directly compared individuals’ experiences of the COVID-19 lockdown with first-time prisoners’ experiences of imprisonment on a range of measures. We found that although people in lockdown (who had never been in prison before) did not necessarily liken lockdown to imprisonment, their subjective experiences of lockdown were comparable to those of first-time prisoners. The pattern of findings was generally consistent when comparing first-time male prisoners with males in lockdown and with females in lockdown. In addition, the findings were fairly similar across the two regions studied (i.e., UK and California). Below, we discuss the main findings, and highlight the strengths and limitations of our approach to understanding psychological experiences of the COVID-19 lockdown, before identifying potential directions for future research.

### Are Experiences of the COVID-19 Lockdown Comparable to Imprisonment?

In some respects, individuals in lockdown demonstrated more positive adjustments to their confinement compared to first-time prisoners, although most of these findings do not necessarily paint a positive psychological picture of lockdown. For instance, it is unsurprising that, unlike prisoners who share a living space with unknown others, some groups in lockdown had more interaction with those they live with (i.e., their family and/or friends). Similarly, although some groups in lockdown felt less unhappy relative to before lockdown than did first-time prisoners before they entered prison, both groups were, nevertheless, less happy than before. Finally, although we found that people in lockdown thought less often about missing their family/friends than did first-time prisoners, some groups in lockdown had a similar frequency of contact with family/friends living elsewhere as did first-time prisoners. Other studies have similarly noted a disruption to social ties during lockdown (e.g., Roy et al., 2020; Sharma and Subramanyam, 2020).

Perhaps the only indicator we found of the COVID-19 lockdown being psychologically better than imprisonment is that, compared to first-time prisoners, people in lockdown thought less often about missing their freedom and some groups in lockdown also thought less often about needing control over their life. Aymerich-Franch (2020) reported that 70.2% of adults in lockdown in Spain felt less free, but did not use a comparison group. The present findings suggest that even if people in lockdown do feel less free, the sense of freedom is still greater than that enjoyed by prisoners. Unlike prisoners, people in lockdown can, for the most part, plan their own daily regime and venture outside their properties for limited exercise and/or essential purposes.

In other respects, however, the experience of lockdown was either similar to, or even worse than, being in prison for the first-time. Females in lockdown in both the UK and California thought about being attacked/beaten up as equally often as did first-time prisoners. Prisons are notoriously violent places (e.g., Blevins et al., 2010), and the COVID-19 lockdown has not only shone a light on the violence that occurs within the home, but also on the rise of such domestic abuse during lockdown (e.g., ABC News, 2020; BBC News, 2020).

We also found that people in lockdown participated in a lesser variety of daily activities than did first-time prisoners. A closer examination of the data showed that whereas over half of first-time prisoners in both regions worked, studied, exercised regularly, and attended a self-help program, the main activities performed by more than half of those in lockdown in these regions were work and exercise. Although we cannot say here whether people in lockdown simply did not engage in other activities such as household chores (Aymerich-Franch, 2020; Chirombe et al., 2020), it is clear that the sorts of activities believed to enrich prisoners’ lives and help them cope with their confinement (e.g., education and self-help programs) were less prevalent in lockdown. The psychological effects of limited engagement in activities during lockdown remain to be seen, although other evidence of people in quarantine has documented feelings of boredom (Brooks et al., 2020).

Finally, and perhaps most concerning, is the finding that people in lockdown felt more hopeless relative to before lockdown compared to first-time prisoners before they went to prison. This supports Ali et al.’s (2020) finding as well as that of Sibley et al. (2020), and is compatible with the growing body of research reporting the mental distress suffered by people in lockdown (Aymerich-Franch, 2020; Odriozola-Gonzalez et al., 2020; Rossi et al., 2020; Srilakshmidevi and Suseela, 2020; White and Van der Boor, 2020). Feelings of hopelessness are predictive of suicide ideation, attempted suicide, and death by suicide (Ribeiro et al., 2018). Calderon-Anyosa and Kaufman (2020) recently found evidence of increased suicides among men in Peru during lockdown, and Caballero-Domínguez et al. (2020) reported increased suicide risk for people in lockdown in Columbia. Others have similarly forecasted increased suicides worldwide (e.g., Sher, 2020; Weems et al., 2020). Thus, the COVID-19 lockdown may have had potentially psychologically devastating effects during the first wave of the pandemic.

Beyond the aforementioned comparison between those in the COVID-19 lockdown and those in prison for the first-time, the present research also found a significant independent effect of age among those in confinement in the UK. On the one hand, older individuals participated in fewer activities and had less social contact with family and friends living elsewhere than their younger counterparts. On the other hand, older individuals were less likely to have negative thoughts pertaining to needing control over their life and missing sex, and were less likely to be accused of (or be charged with) rule-breaking than younger individuals. These latter findings are compatible with studies of adults in lockdown in Italy, India, and Spain which also report that younger people demonstrate more adverse or negative psychological outcomes (Aymerich-Franch, 2020; Rossi et al., 2020; Singhal and Vijayaraghavan, 2020). Later, we consider the psychological trajectory that older people in lockdown may find themselves on.

### Strengths and Limitations

There have been calls for research on the psychological impact of the Covid-19 (SARS-CoV-2) pandemic (e.g., Verger et al., 2020). By directly comparing individuals in lockdown with first-time prisoners on a wide variety of responses (including behavioral, social, thoughts and emotions), and after controlling for demographic differences between the two groups, the present research provides a way of contextualizing and interpreting a range of psychological effects of lockdown. It is reasonable to assume that, as a form of confinement, imprisonment is (and should be) worse than lockdown, thus demonstrating that people in lockdown feel the same or worse than first-time prisoners is insightful. The fact that these findings were observed in two different regions emphasizes their generalizability and robustness.

Nonetheless, there are some potential limitations of our approach. First, there is a large time gap between the two sources of data (i.e., prison and lockdown). In order to avoid the confounding effect of the COVID-19 outbreak in the prison system, we opted to use data that had been collected from prisoners before the pandemic. Our search for such data focused on studies that included a range of quantitative measures of prisoner adaptation. There is mixed evidence as to whether prison environments have improved or deteriorated over the intervening years (see Prison Reform Trust, 2020), and it is unclear if, and how, such changes would affect first-time prisoners’ subjective experiences.

Second, we compared females in lockdown to first-time, male prisoners. It is therefore, unclear if females in lockdown fare better or worse than first-time, female prisoners. According to Kruttschnitt and Gartner’s (2003) review of research on women’s imprisonment, female responses to imprisonment are similar to those found in male prisoners, although women tend to be more active in choosing their patterns of adjustment.

Third, we found that whereas people in lockdown thought less often about missing sex than did first-time prisoners, who are deprived of heterosexual relations, our study does not capture the longing for homosexual relations that have been identified in some recent research on the COVID-19 lockdown (Sharma and Subramanyam, 2020).

Fourth, at the time of data collection, the lockdown sample had spent considerably less time in confinement than the prison sample, and so they may not have had sufficient time to adapt to their situation. However, Dhami et al.’s (2007) survey of 712 adult, male US federally sentenced prisoners in three prisons (high, medium and low security), found that after controlling for sentence length and prison security level, time spent in prison was only predictive of some of the variables measured in the present research. Specifically, time spent in prison was negatively associated with disciplinary infractions and positively associated with feelings of hopelessness and thoughts about needing control over one’s life. This suggests that over time, people in lockdown may continue to feel more hopeless than before, and their frequency of thoughts about needing control over their life (which are currently less than first-time prisoners) may increase.

### Directions for Future Research

Since conducting the present research, most Governments, including those in the UK and US have begun to ease lockdown restrictions. However, it is widely believed that there will be other waves of COVID-19 (Wise, 2020). If strict lockdown policies are re-imposed, then future research could explore whether people are better able to cope with lockdown. This could be done by comparing their responses to those of prisoners who have prior prison experience. Recurrent prisoners differ in their adjustment to confinement compared to first-time prisoners. For instance, whereas recurrent prisoners may demonstrate some positive adjustments such as greater psychological wellbeing and more participation in self-help programs (e.g., Souza and Dhami, 2010), they may also demonstrate some negative behaviors such as rule-breaking (e.g., Bosma et al., 2020). In the present study, people in lockdown were either more or equally compliant with the rules of lockdown as were first-time prisoners with the rules of prison, however, there may be less compliance during future lockdowns.

The fact that the isolation or shielding of those particularly vulnerable to the more severe consequences of COVID-19 such as the elderly is likely continue after any lockdown ends and perhaps until a vaccine is available, makes it imperative to understand the psychological trajectory that such individuals may find themselves on. Future research could examine if older people respond to lockdown in the same ways as older prisoners do. For instance, Maschi et al. (2015) reported that a lack of social contact can be a major source of stress and trauma for prisoners over the age of 50. In the present study, older individuals had less social contact with family and friends living elsewhere than their younger counterparts, and over time, this could serve to reduce their psychological well-being.

Finally, future research on the COVID-19 lockdown could explore factors that may predict individual’s patterns of adjustment. Prison researchers have examined the independent, relative and interactive effects of a range of pre-prison and in-prison factors in predicting adaptations to imprisonment (e.g., Dhami et al., 2007; Dye, 2010; DeLisi et al., 2011). In the context of the COVID-19 lockdown, this would mean, for example, measuring the extent to which factors such as quality of life before and current living conditions predict adjustment to lockdown. Patterns of adjustment can have implications for how well individuals readjust to life after lockdown restrictions end, and so the findings of such research can identify those who may require support to help them readjust.

## Conclusion

The present research sheds some light on the psychological, emotional, social and behavioral responses to the COVID-19 lockdown among adults in the UK and California relative to another form of confinement (i.e., imprisonment). Lockdown and imprisonment can be compared on several dimensions, and despite the apparent differences, the present research demonstrates that psychological parallels can be drawn between these two forms of confinement. Thus, although Governments do not intend to punish their citizens, lockdown policies may have unintended adverse consequences.

## Data Availability Statement

The datasets presented in this study can be found in online repositories. The names of the repository/repositories and accession number(s) can be found below: https://osf.io/8gvmk/.

## Ethics Statement

The studies involving human participants were reviewed and approved by City Research Ethics Committee. Participants provided their written informed consent to participate in this study.

## Author Contributions

MD contributed to research design, data analysis, interpretation, and intellectual content. LW-C contributed to data collection, preparation of data, data analysis, and intellectual content. PA contributed to research design, interpretation, and intellectual content. All authors contributed to the article and approved the submitted version.

## Conflict of Interest

The authors declare that the research was conducted in the absence of any commercial or financial relationships that could be construed as a potential conflict of interest.

## Footnotes

1 Police were given powers to enforce the lockdown in the UK which included fines of up to £60 for a first violation (UK Government, 2020c). Violation of the Californian lockdown is a misdemeanor which is punishable by a fine of up to $1,000 or imprisonment of up to 6 months or by both (Executive Department State of California, 2020).

2 Imprisonment can vary considerably across the world (see Prison Insider, 2020). For present purposes, the focus is on Anglo-American jurisdictions because that is the focus of the present research.

3 In California, the “stay at home” order was issued on March 19^*t**h*^, and no initial time period was provided. The California State Department of Public Health (2020) issued an order on May 7^*t**h*^ easing some lockdown restrictions. In both the UK and California, some regions (e.g., Scotland and Los Angeles, respectively) announced that they would ease lockdown restrictions at a slower pace.

4 https://osf.io/8gvmk/

5 https://osf.io/akvqd

6 Dhami et al.’s (2007) study also included samples of high and low security Federal prisoners in California. These are not included in the present analysis because we wanted to keep prison security level constant across the two regions studied (Souza and Dhami’s (2010) study only focused on medium security prisoners).

7 Dhami et al. (2007) had five categories of 13 dependent variables, but these included an item asking about interaction with prison guards, which is irrelevant for our lockdown samples. In addition, whereas Dhami et al. (2007) counted the number of programs that prisoners participated in as a separate dependent variable, we simply added the binary version of it (participated in programs v. not) to the aggregated activity variable.

8 There were two items asking about work for the lockdown samples (see Supplementary Table A1), and an affirmative response to either one was used here.

9 There were three items asking the prison samples about contact with family/friends from the outside (see Supplementary Table A1), and we averaged responses on these to create one variable.

10 The wording for this item was different for the lockdown samples (see Supplementary Table A1) because we considered that a question asking about being charged of breaking lockdown rules by the police would be too restrictive.

11 Data from some participants in the two lockdown samples was excluded from further analysis because they had been in prison before (UK: *n* = 1, California: *n* = 5).

12 To aid statistical analysis, we performed a median split on the time spent in lockdown/prison covariate.

13 In order to ease the flow of reading, from this point forward, only the statistically significant covariates will be identified.

## Appendix

**TABLE A1:** Items and response scales used in present analyses (scale size and coding of variables shown in brackets).

Prison samples	Lockdown samples
Do you have a job in here? Yes (1)/No (0)	Are you working from home? Yes (1)/No (0) Are you going out to work? Yes (1)/No (0)

Do you take education classes in here? Yes (1)/No (0)	Are you studying from home? Yes (1)/No (0)

How often do you go to the gym to workout/exercise in here? Never (1) –∣ – rarely –∣ – sometimes –∣ – often –∣ – always (9)	How often do you exercise? Never (1) –∣ – rarely –∣ – sometimes –∣ – often – ∣ – always (9)

How often do you take part in religious activities in here? Never (1) –∣ – rarely –∣ – sometimes –∣ – often – ∣ – always (9)	How often do you take part in religious/spiritual activities? Never (1) –∣ – rarely –∣ – sometimes –∣ – often –∣ – always (9)

Have you attended in programs in here? Yes (1)/No (0)	Have you started any self-improvement/self-help programs or hobbies while in lockdown? Yes (1)/No (0)

How often do you think of the following in prison? Missing your freedom: Needing control over life: Missing having sex: Missing your family/friends: Being attacked/beaten up: Responses to each question were provided on the following scale: Never (1) –∣ – rarely –∣ – sometimes –∣ – often –∣ – always (9)	How often do you think of the following in prison? Missing your freedom: Needing control over life: Missing having sex: Missing your family/friends: Being attacked/beaten up: Responses to each question were provided on the following scale: Never (1) –∣ – rarely –∣ – sometimes –∣ – often –∣ – always (9)

How much do you mix with other inmates in prison? Not at all (1) – –∣ – –∣ – – somewhat – –∣ – –∣ – – A lot (7)	How much do you mix with others you live with? Not at all (1) – –∣ – –∣ – – somewhat – –∣ – –∣ – – A lot (7)

How often do your friends/family from outside visit you in here? How often do your friends/family from outside write to you in here? How often do you telephone your friends/family? Responses to each question were provided on the following scale: Never (1) –∣ – rarely –∣ – sometimes –∣ – often –∣ –constantly (9)	How often do your friends/family living elsewhere contact you while you are in lockdown? Never (1) –∣ – rarely –∣ – sometimes –∣ – often –∣ –constantly (9)

How have you been feeling lately in here? Happy: Hopeless: Responses to each question were provided on the following scale: Much less (1) –∣ –∣ –∣ – same as before –∣ –∣ –∣ – Much more (9)	How have you been feeling lately in lockdown? Happy: Hopeless: Responses to each question were provided on the following scale: Much less (1) –∣ –∣ –∣ – same as before –∣ –∣ –∣ – Much more (9)

How often have you been charged with misconduct in here by the guards/warden? Never (1) – –∣ – –∣ – – sometimes – –∣ – –∣ – – Often (7)	How often have people accused you of disobeying the rules of lockdown? Never (1) – –∣ – –∣ – – sometimes – –∣ – –∣ – – Often (7)

How much of this sentence have you now served? Years, Months, Weeks, Days	How many weeks have you spent in lockdown so far? I’m not in lockdown, 1….More than 12 weeks

How old are you?	How old are you?

	How do you identify? Male/Female/Other

How would you describe your ethnic group? White/Hispanic/Black/Asian/Other	How would you like to describe yourself? White/Hispanic or Latino American/Black or African American/Asian from Indian subcontinent/Asian from Far East/Southeast Asia/Mixed race/Other

What is the highest level of education you completed before prison? Did not finish high school/Finished high school/Took some college or university/Finished college or university	What is the highest level of education you completed? Did not finish high school/Finished high school/Took some college or university/Finished college or university

What kind of job did you have before prison? Security/Sales or clerical/Laborer/Unemployed/Student/Retired/Other	What was your employment status before lockdown? Employed/Student/Retired/Unemployed

What kind of relationship were you in before coming to prison? Married/Girlfriend/Single/Divorced or separated/Widowed	What kind of relationship were you in before lockdown? Married or civil partnership/Partner or cohabiting/Single/Divorced or separated/Widowed

How often did you take drugs before prison? Never (1)– –∣ – –∣ – – sometimes – –∣ – –∣ – – Often (7)	How often did you take illegal drugs before lockdown? Never (1) – –∣ – –∣ – – sometimes – –∣ – –∣ – – Often (7)
